# Therapeutic Potential of a New Jumbo Phage That Infects *Vibrio coralliilyticus*, a Widespread Coral Pathogen

**DOI:** 10.3389/fmicb.2018.02501

**Published:** 2018-10-24

**Authors:** Loïc Jacquemot, Yvan Bettarel, Joanne Monjol, Erwan Corre, Sébastien Halary, Christelle Desnues, Thierry Bouvier, Christine Ferrier-Pagès, Anne-Claire Baudoux

**Affiliations:** ^1^Sorbonne Universités UPMC Paris 06, CNRS, UMR7144 Adaptation et Diversité en Milieu Marin, Station Biologique de Roscoff, Roscoff, France; ^2^MARBEC, Université Montpellier, IRD, CNRS, Ifremer, Montpellier, France; ^3^Sorbonne Universités UPMC Paris 06, CNRS, FR2424 Fédération de Recherche, Station Biologique de Roscoff, Roscoff, France; ^4^Aix Marseille Université, Microbes, Evolution Phylogeny and infection (MEPHI), CNRS FRE2013, IRD 198, AP-HM, IHU - Méditerranée Infection, Marseille, France; ^5^Centre Scientifique de Monaco, Equipe Ecophysiologie Coralienne, Monaco, Monaco

**Keywords:** phage therapy, coral disease, *Vibrio coralliilyticus*, viral genomics, phage–host interactions

## Abstract

Biological control using bacteriophages is a promising approach for mitigating the devastating effects of coral diseases. Several phages that infect *Vibrio coralliilyticus*, a widespread coral pathogen, have been isolated, suggesting that this bacterium is permissive to viral infection and is, therefore, a suitable candidate for treatment by phage therapy. In this study, we combined functional and genomic approaches to evaluate the therapeutic potential of BONAISHI, a novel *V. coralliilyticus* phage, which was isolated from the coral reef in Van Phong Bay (Vietnam). BONAISHI appears to be strictly lytic for several pathogenic strains of *V. coralliilyticus* and remains infectious over a broad range of environmental conditions. This candidate has an unusually large dsDNA genome (303 kb), with no genes that encode known toxins or implicated in lysogeny control. We identified several proteins involved in host lysis, which may offer an interesting alternative to the use of whole bacteriophages for controlling *V. coralliilyticus*. A preliminary therapy test showed that adding BONAISHI to an infected culture of *Symbiodinium* sp. cells reduced the impact of *V. coralliilyticus* on *Symbiodinium* sp. photosynthetic activity. This study showed that BONAISHI is able to mitigate *V. coralliilyticus* infections, making it a good candidate for phage therapy for coral disease.

## Introduction

Coral reefs are one of the most productive and diversified ecosystems on the planet (Connell, 1978) and they provide a wealth of ecological services as well as being economically important, supporting fisheries, tourism, and medical applications (Moberg and Folke, 1999; Hughes et al., 2003; Cooper et al., 2014). The health of these ecosystems is severely threatened by the combined effect of local anthropogenic pressures and global changes (Jackson et al., 2001; Hughes et al., 2003; Pandolfi et al., 2003; Bellwood et al., 2004). Over-exploitation of marine species, pollution, and increased sea surface temperature are associated with the emergence of coral diseases, which are contributing to the decline of coral reefs worldwide.

Several studies have identified *Vibrio* spp. (⋎-Proteobacteria*)* as causative agents of coral bleaching for multiple coral species and in multiple locations (Ushijima et al., 2014; reviewed in Mera and Bourne, 2018). *Vibrio coralliilyticus* (*V. coralliilyticus*) has emeregd as an important bacterial pathogen model for understanding the establishement and propagation of coral disease (Sussman et al., 2008; O'Santos et al., 2011; Garren et al., 2014; Pollock et al., 2015). Studies have shown that V. *coralliilyticus* infection is temperature dependent and infects the coral endosymbiont *Symbiodinum* through the production of proteases that inhibit photosynthesis. This results in the loss of the endosymbiont from the coral tissues and ultimately leads to coral bleaching (Ben-Haim et al., 2003; Sussman et al., 2008, 2009; Cohen et al., 2013). With the increasing devastation of coral reefs, the development of new tools and strategies to control pathogens and treat diseased corals is becoming a major issue. Currently, biocontrol strategies, such as phage therapy, are being seriously evaluated for mitigating coral diseases (Efrony et al., 2009; Teplitski and Ritchie, 2009; Atad et al., 2012; Cohen et al., 2013).

The potential of viruses (more specifically bacteria viruses also referred to as bacteriophages or phages) as therapeutic agents to treat infectious diseases has been known for a long time (d'Herelle, 1926; Duckworth, 1976; Duckworth and Gulig, 2002). The idea of phage therapy arose from the early discovery that a given virus usually infects a single host species, leaving the rest of the microbial community untouched. Moreover, viruses are obligate intracellular organisms and, therefore, their production is self-regulated and limited by the availability of suitable hosts. Over the past decade, there have been promising *in vitro* and *in situ* trials of phage therapy for corals. One used BA3, a virulent bacteriophage that infect the causative agent of white plague disease (Efrony et al., 2007, 2009), to inhibit the progression of the disease and its transmission to healthy corals (Atad et al., 2012). Although this research is still at an early stage, this promising result in the open sea suggest that *in situ* phage therapy for coral diseases is achievable (Atad et al., 2012). The isolation and characterization of new bacteriophages is, therefore, essential to increase the collection of potential candidates for therapeutic assays.

The therapeutic value of a candidate bacteriophage relies on the characterization of viral properties such as the virion stability, growth kinetics, viral yield, and host range. Understanding the lifestyle of the candidate phage is probably the key to their use in therapy. Only virulent bacteriophages, which replicate through a lytic cycle and kill their host after infection, will be suitable candidates. Temperate bacteriophages, which replicate using a lysogenic cycle, may improve host fitness through gene transfer. It is, however, difficult to distinguish between virulent and temperate phage because temperate viruses can switch to a lytic cycle in response to environmental changes, such as temperature, pH salinity, UV, pollution, or nutrient availability (Jiang and Paul, 1996; Williamson and Paul, 2006, reviewed in Howard-Varona et al., 2017). Furthermore, infection dynamics can be highly variable, even between two closely related hosts (Holmfeld et al., 2014; Dang et al., 2015). Over the past decade, genomics has greatly improved our understanding of host—virus interactions, and is the key to establishing whether a candidate is an obligate lytic bacteriophage (Howard-Varona et al., 2017). Bacteriophage genome sequencing is also essential for evaluating the safety of a candidate (absence of toxins and temperate phage hallmarks), and to provide information on the candidate's evolution history and ecology (Lobocka et al., 2014).

In this study, we report a novel bacteriophage, hereafter referred to as *Vibrio* phage BONAISHI that infects the model coral pathogen *V. coralliilyticus*. We studied it using a combination of functional, genomic, and metagenomic approaches to evaluate the potential of this phage for mitigating disease caused by *V. coralliilyticus*.

## Material and methods

### Virus isolation

Seawater samples were collected from coral surrounding water off Whale Island (Van Phong Bay, Vietnam). A 50 mL aliquot was supplemented with 10% (v/v) Marine Broth (MB, Difco) and the mixture was enriched with 1 mL *Vibrio coralliilyticus* LMG20984 (YB1) culture and incubated for 48 h at 25°C (Brussaard et al., 2016). The sample was clarified (7,000 g, 15 min) and the supernatant was filtered through 0.2 μm PES filters to separate the viral community. A 100 μL aliquot of the filtrate was added to 900 μL YB1 culture and incubated for 30 min at 25°C. The mixture was included in molten agar (Marine Broth supplemented with 0.6% noble agar) and spread on a Marine Agar plate. After 48 h incubation, a translucent plaque indicating host lysis was removed from the bacterial lawn, eluted in 0.22 μm filtered Salt Marine (SM) buffer (100 mM NaCl, 8 mM MgSO4, 50 mM Tris-HCl pH 8.0) and combined with a host culture in a plaque assay (Brussaard et al., 2016). This procedure was repeated twice to ensure the clonality of the bacteriophage. Finally, the clonal phage suspension and host culture were used in a plaque assay giving confluent lysis. The plaques were eluted in SM buffer, the eluent was clarified by centrifugation (7,000 g, 30 min, 4°C), and the supernatant was filtered through 0.22 μm and stored at 4°C until use.

### Transmission electronic microscopy

A 10 μL aliquot of the phage suspension was loaded onto a Formvar/carbon film coated 400 mesh copper grid (Euromedex). After 5 min incubation, the grid was blotted with filter paper and stained with 2% uranyl acetate for 30 s, blotted again to remove excess dye and air dried for 30 min (Ackermann and Heldal, 2010). The specimen was imaged using JEOL 1400 transmission electron microscope operating at 120 keV at a magnification of 80,000X.

### Environmental range of infectivity

The tolerance of BONAISHI to temperature and pH ranges was evaluated by monitoring the loss of infectivity of a freshly produced suspension by spot test. For evaluating the temperature range, 100 μL of viral suspension (5 x 10^8^ PFU/mL) was incubated at temperatures from 4 to 70°C for 30 min in a dry bath. The samples were then cooled for 5 min at 4°C and virus infectivity was assessed by spot test. Briefly, 5 μL of the treated viral suspension was spotted on a host lawn obtained by plating a 1:4 mixture of host culture in molten agar onto a marine agar plate. For evaluating the pH range, 100 μL of viral suspension were added to 900 μL SM buffer adjusted at pH ranging from 2 to 10. The samples were incubated for 24 h at 4°C and then spot-tested as described above.

### Host range

A selection of 43 bacterial strains (Table S1) related to the original host *V. coralliilyticus* YB1 were used to determine the host specificities of BONAISHI. The ability of BONAISHI phage to infect these strains was determined by pairwise infection. The bacterial strains were grown in Marine Broth media (DIFCO) overnight. Each culture was included in molten agar (Marine Broth supplemented with 0.6% noble agar) and spread on a Marine Agar plate. A freshly produced suspension of BONAISHI was serially diluted in SM buffer and a 5 μL drop of each dilution was spotted on a bacterial lawn. After 24–48h incubation at 25°C, the formation of translucent spots, indicative of host lysis, was recorded.

### Life strategy

The growth cycle of BONAISHI was tested on two *V. coralliilyticus* strains including the original host LMG 20984 (YB1) and the alternate host LMG 23696 (P1) (Middelboe et al., 2010). Both host cultures were grown in MB and divided into four 25 mL sub-cultures. One sub-culture served as control and the remaining 3 were inoculated with a freshly produced BONAISHI suspension at multiplicity of infection (MOI) of 0.1 as determined by flow cytometry (see below). All cultures were incubated at 25°C for 48 h. Samples for viral and bacterial counts were taken every hour for 10 h, and then every 4 h for 48 h. Samples were immediately fixed with glutaraldehyde (0.5% final concentration) for 10 min at 4°C, flash frozen in liquid nitrogen, and stored at −80°C until flow cytometry analysis (see below). Bacterial host and virus counts were used to calculate the phage latent period and burst size. The latent period corresponds to the time elapsed between the viral inoculation and the release of virions. The burst-size, which corresponds to the number of virions produced per infected host cell, was determined by the ratio of the net increase in virus concentration over the net decrease in host concentration during the first burst.

### Flow cytometry

For determining the bacterial abundance, samples were diluted in autoclaved 0.2 μm filtered TE buffer (10 mM Tris-HCl, 1 mM EDTA, pH 8.0) and stained with a SYBR Green (10,000-fold dilution of commercial solution) for 15 min in the dark at ambient temperature. For determining the viral abundance, 100–1,000-fold diluted samples were stained with SYBR Green (20,000-fold dilution of commercial solution) for 10 min in the dark at 80°C. Analyses were performed using a FACS Canto II equipped with an argon-laser (455 nm). The trigger was set on the green fluorescence and the sample was delivered at a rate of 0.06 mL min^−1^ and analyzed for 1 min (Brussaard, 2004). Viral and bacterial counts were corrected for a blank consisting of TE-buffer with autoclaved 0.2 μm filtered seawater at the corresponding dilution.

### Genome extraction and sequencing

A 1 L viral suspension was concentrated by ultrafiltration using a 30 kDa PES membrane (Vivaflow 50, Vivascience) and centrifugal concentrator (Vivaspin 20, 30 kDa, PES) to a final volume of 2 mL. The concentrate was subsequently purified on linear sucrose gradient (10–40 % in 0.2 μm SM) by ultracentrifugation (SW41.Ti rotor, 96,808 g, 30 min at 4°C). The BONAISHI particles formed a well resolved band that was extracted, dialyzed against SM buffer using a centrifugal concentrator (Vivaspin 20, 30 kDa, PES) and stored at 4°C until use. The genome of the purified phage suspension was extracted using a DNAeasy Blood and Tissue kit (QUIAGEN, Valencia, CA) according to the manufacturer's protocol. Samples were sent to GATC Biotech, and sequenced using PACBIO RS II (17,131 mean read length). Raw read sequences assembled as a single contig using HGAP software (Chin et al., 2013). The final draft assembly was 303, 340 bp with an average coverage of 1,720x and an average base quality score of 86%.

### Bioinformatic analysis

#### Phage genome annotation

Putative coding DNA sequences (CDS) in the BONAISHI genome were predicted using Glimmer (Delcher et al., 1999) and Genemark (Besemer et al., 2001). The coordinates of each translated open reading frames (ORF) were also inspected manually. ORF smaller than 200 base pairs (bp) were removed from the analysis. The predicted amino acid sequences were assigned manually by BLASTP and PSI-BLAST (cutoff e-value < 10^−5^) against the NCBI non-redundant database (January 2018) and InterProScan 5 (Jones et al., 2014), as well as the fully automated RAST server annotation service (Aziz et al., 2008). BLASTP was used to search for putative toxins in the databases MvirDB (Zhou et al., 2006), VirulenceFinder (Chen et al., 2005), Vibrio-base (Choo et al., 2014), and t3DB (Lim et al., 2009; Wishart et al., 2015) toxin databases. The IntegrallDB (Moura et al., 2009) and ACLAME (Leplae et al., 2006) databases were used to check for prophage-like sequences. tRNAScanSE (Lowe and Eddy, 1997) and Aragorn (Laslett and Canback, 2004) were used to check for tRNA. The genome map was produced using Artemis (Rutherford et al., 2000) and DNA Plotter (Carver et al., 2009). The BONAISHI genome sequence has been submitted to the GenBank database under accession number MH595538.

#### Terminase large subunit (TerL) protein phylogeny

The amino acids sequence of the terminase large subunit from 63 jumbo phages including BONAISHI were used for phylogenetic analysis. Sequences were trimmed to 406 bp, the minimum sequence length of *Aeromonas* phage px29, using BioEdit (Hall, 1999). Sequences were aligned by Muscle and the tree was constructed by Maximum Likelihood with 1,000 bootstrap iterations using Mega 6.06 (Tamura et al., 2013).

#### Host genome analysis

The clustered regularly interspaced short palindromic repeats (CRISPR), in the pathogenic *V. coralliilyticus* strains P1 and YB1 genome, were searched for genetic signatures of viral resistance mechanisms using CRISPRfinder (Grissa et al., 2007). Genetic exchange between the phage BONAISHI and its bacterial hosts was checked by homology between the phage ORFs and the ORFs of *V. coralliilyticus* P1 (AEQS00000000) and YB1 (ACZN00000000) using BLASTP.

#### Metagenomic analysis

To determine the distribution of BONAISHI, the genome was used to recruit homologous reads from 56 coral-associated virome in Metavir (Roux et al., 2011), and 137 CAMERA Broad Phage metagenomes (Seshadri et al., 2007) and viral contigs in IMG/VR (Paez-Espino et al., 2017, Table S2). These samples comprised a wide range of marine environments including tropical and temperate pelagic ecosystems, healthy and diseased coral reefs including slurry from individual coral colonies, coral mucus, and the water from coral reefs. We also carried out recruitments in prokaryote metagenome from 4 coral atolls (Dinsdale et al., 2008) to check whether BONAISHI genome sequences were inserted into prokaryote genomes. Each reads served as a query and was assigned to a (single) best-matching hit by BLASTN and TBLASTX if the alignments met the following criteria: e-value < 10^−3^, alignment length > 50, bitscore > 40. BLASTN parameters were set to: open gap cost = 8, extend gap cost = 6, match reward = 5, mismatch penalty = −4, word size = 8. Reads were recruited from each metagenome in order to determine the fraction of recruited reads that can be assigned to BONAISHI.

### Preliminary treatment of diseased symbiodinium

Culture of *Symbiodinium* sp. cells (Clade A1) originally extracted from the scleractinian coral *Galaxea fascicularis* (Goiran et al., 1996) were maintained in the laboratory in F/2 medium (Guillard and Ryther, 1962) prepared from Guillard's Marine Water Enrichment Solution (Sigma-Aldrich G9903). Cultures were maintained at 25°C under 100 μmol photons m^−2^ s^−1^ of white light provided by fluorescent tubes (Mazda 18WJr/865) using a 12:12 light:dark cycle. One day prior to the therapy assay, exponentially growing *Symbiodinium* sp. cultures were transferred to 30°C under the same light conditions. A 20 mL aliquot was concentrated at 5,000 g for 10 min at 30°C (VIVASPIN 20, PES, 30 kDa). The retentate was gently resuspended in 20 ml EDTA free F/2 medium and the procedure was repeated twice to wash the culture. The *Symbiodinium sp*. abundance was determined by flow cytometry and adjusted to 10^4^ cells mL^−1^. *V. coralliilyticus YB1* was cultured overnight in MB and then purified in the same way. Bacterial abundance was determined by flow cytometry and adjusted to 10^7^ cells mL^−1^. A freshly produced suspension of BONAISHI was purified by sucrose gradient and diluted in SM buffer. The viral abundance was determined by flow cytometry.

For the therapy assay, the algal culture was split into 3 equal sub-cultures. One sub-culture served as control, while two of the subcultures were inoculated with an equal volume of *V. coralliilyticus YB1*. Of these, one was also inoculated with 10^8^ phages mL^−1^. All three treatments were sampled at 0, 5, 20, and 60 min to determine the photosystem II quantum yield of *Symbiodinium* sp. cells using a pulse amplitude modulated fluorimeter (Phyto-PAM, Walz) connected to a chart recorder (Labpro, Vernier). After 5 min relaxation in darkness, the non-actinic modulated light (450 nm) was turned on in order to measure the fluorescence basal level, F_0_. A saturating red light pulse (655 nm, 4 000 μmol quanta m^−2^ s^−1^, 400 ms) was applied to determine the maximum fluorescence level in the dark adapted sample, F_M_. The maximal photosystem II fluorescence quantum yield of photochemical energy conversion, F_V_/F_M_, was calculated using the following formula:

(1) $$\begin{array}{l} \frac{F_{V}}{F_{M}}=\frac{\left(F_{M}-F_{0}\right)}{F_{M}} \end{array}$$

## Results

### Morphology

**Figure 1:**
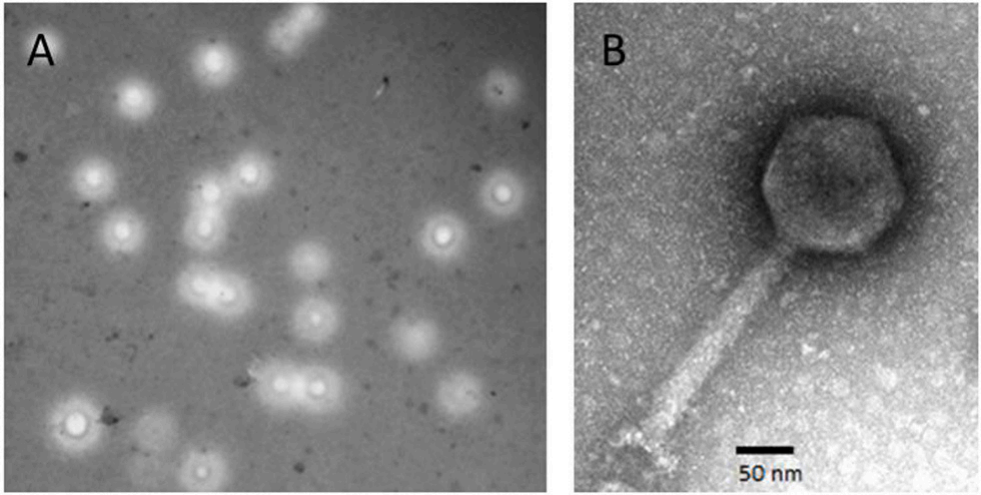
Morphology of Vibrio phage BONAISHI. **(A)** BONAISHI forms large, round plaque on a lawn of *V. coralliilyticus YB1 on* 0.6% soft agar. **(B)** Transmission electron micrographs of a negatively stained particle of bacteriophage BONAISHI. The icosahedral head (120 nm in diameter) and the long, contractile tail (190 nm in length) suggest that BONAISHI belongs to the *Myoviridae* family.

The *Vibrio* phage BONAISHI formed relatively large, round plaques on *V. coralliilyticus YB1* and produced high titer suspension. TEM microscopy showed that BONAISHI has an isometric capsid of 120 nm in diameter connected to a 190 nm long, contractile tail (Figure 1). This indicates that BONAISHI belongs to the order of the *Caudovirales* and the family of the *Myoviridae*.

### Tolerance to environmental factors

The incubations showed that BONAISHI can tolerate a pH ranging from 3 to 10 and temperatures ranging from 4 to 50°C without loss of infectivity (Table 1). This high tolerance suggests that the phage is very stable in the environment.

**Table 1:** Tolerance of *Vibrio* phage BONAISHI to temperature and pH.

**Treatments**	**Infectivity**
**TEMPERATURE (**°**C)**	
4	+
15	+
20	+
25	+
30	+
35	+
40	+
45	+
50	+
60	-
70	-
**pH**	
2	-
3	+
4	+
5	+
6	+
7	+
8	+
9	+
10	+

### Host specificities

Spot tests using a broad range of potential hosts indicated that BONAISHI was able to infect and lyse several strains of *V. coralliilyticus* of interest (Table S1). Besides *V. coralliilyticus* YB1, BONAISHI can infect another known coral pathogen, *V. coralliilyticus* P1, as well as *V. coralliilyticus* LMG21348, isolated from a bleached coral colony (*Pocillopora damicornis*), and *V. coralliilyticus* 1H13, isolated from the mucus of a *Fungia* specimen. This phage did not lyse of any of the closely related species in the test, suggesting that it is species-specific.

### Life cycle

**Figure 2:**
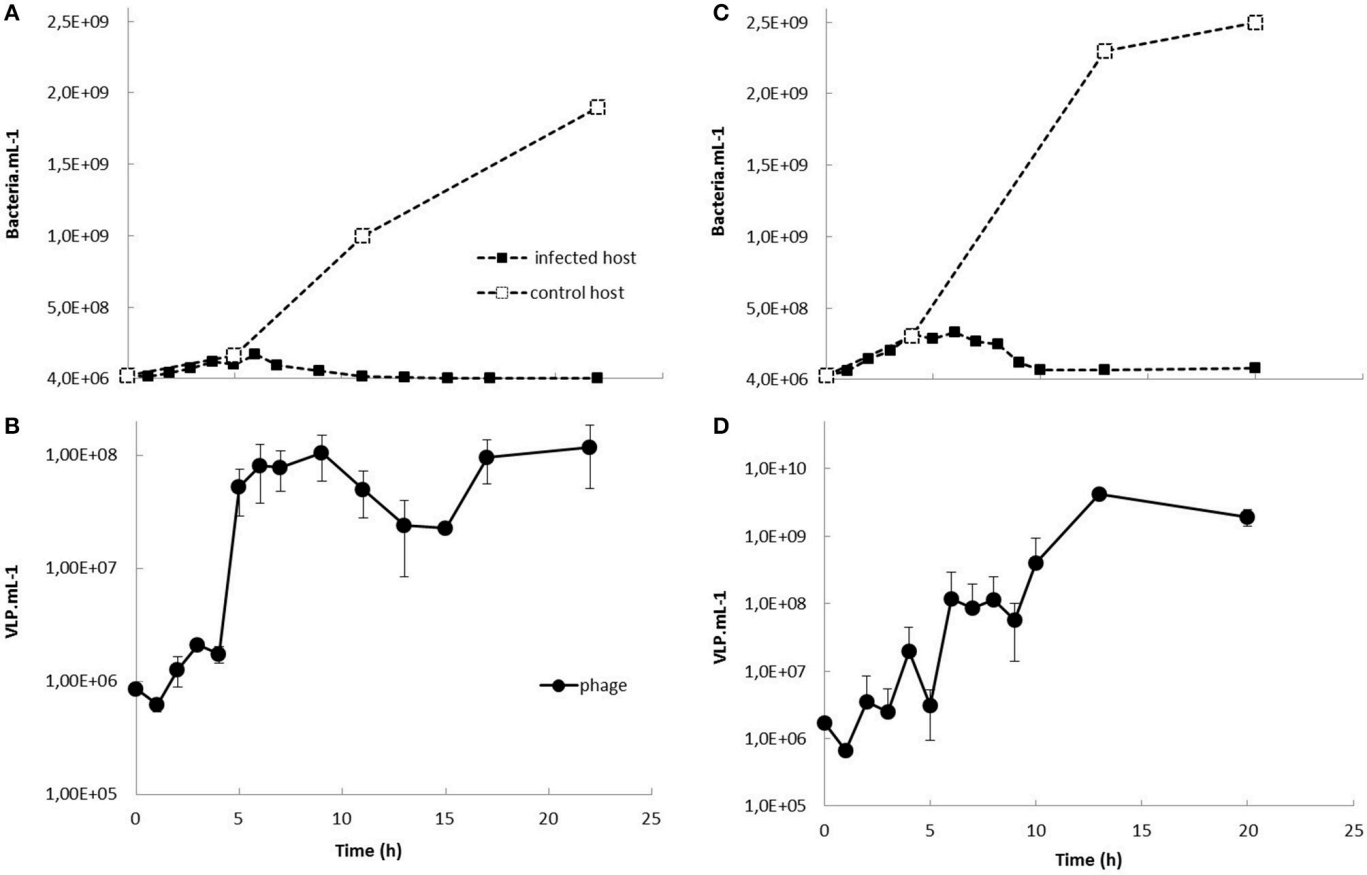
One-step growth experiments of BONAISHI on *Vibrio coralliilyticus* strain P1 **(A,B)** and YB1 **(C,D)**. Bacteria and bacteriophage counts were assessed by flow cytometry upon staining with the nucleic acid dye SYBR Green (Life technology) according to Brussaard (2004).

One-step growth experiments showed that the phage readily propagated on each of its hosts with a latent period of 2–3 h (strains P1 and YB1) and burst size of 8 (P1) and 19 (YB1) (Figure 2). Nearly all the virions produced (96%) were infectious virions per infected cell. The infected host culture collapsed rapidly and there was total lysis 10 h after inoculation.

### General features of BONAISHI genome

**Figure 3:**
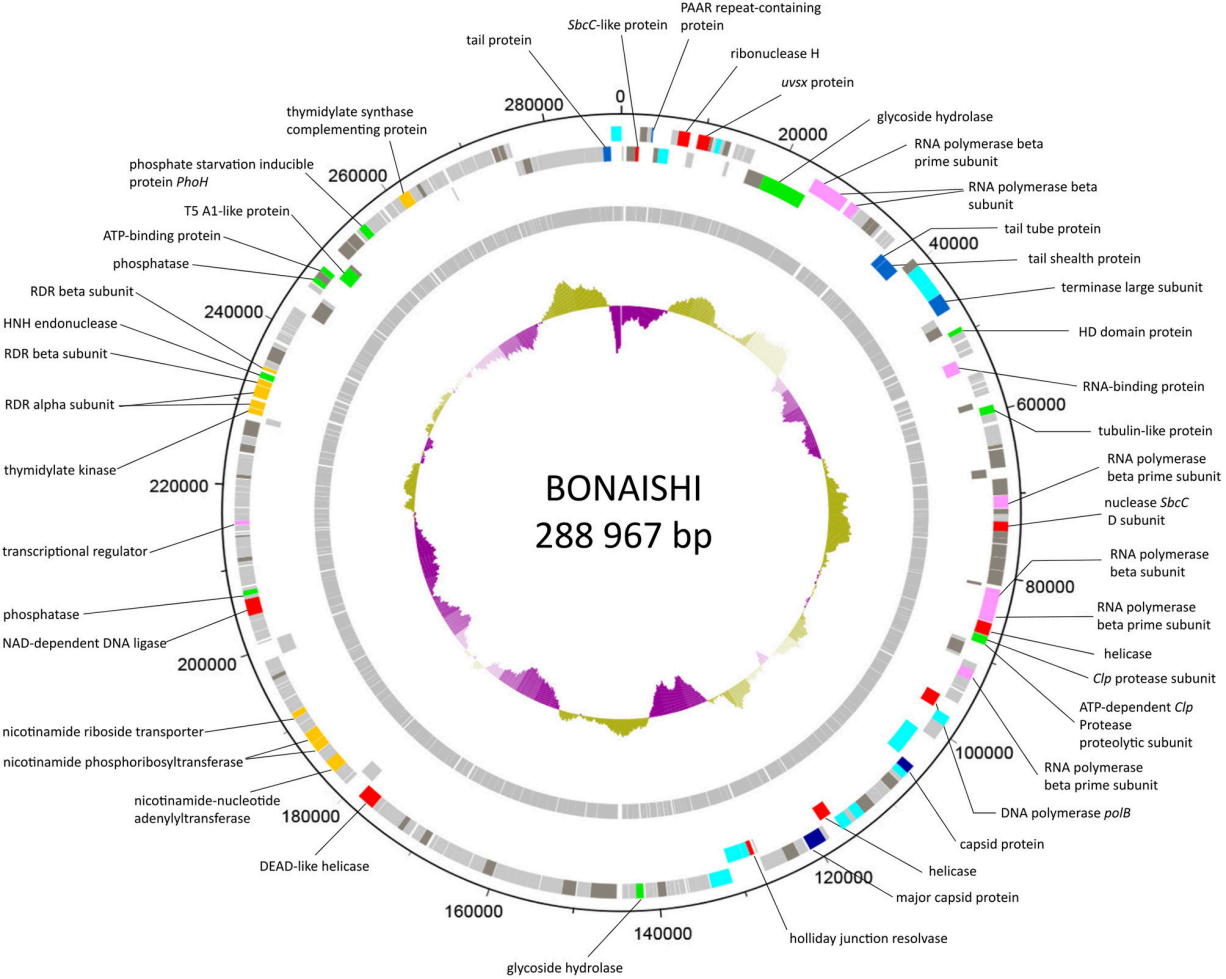
Genome map of BONAISHI. The 110 protein-coding genes are shown as colored blocks. Protein-coding genes that are transcribed in the forward and reverse direction are located on the external and internal circle, respectively. The functional assignments of the protein-coding genes are derived from blastp matches in NCBI nr and expert databases. We confidently discriminated proteins involved in general virion structure (light blue), head, and tail structure and assembly (dark blue), DNA replication,recombination and repair (red), nucleotide metabolism (orange), DNA transcription (pink), and host phage interactions (green). Genes marked “h.p.” made strong matches to hypothetical protein sequences in the database with no identified functions (dark gray). The GC content of the genome sequence is indicated by the internal purple or green histograms.

The genome of BONAISHI consists of a large double-stranded circularly permuted DNA sequence of 303,340 base-pair (bp) with a % G+C content of 42.5% (Figure 3). Terminal duplications at the extremities of the assembled sequence are 14,373 bp, giving a non-redundant genome of 288,967 bp. Glimmer and Genemark predicted 301 putative ORFs, which comprised 93.8% of the total sequence and were mostly oriented in a single direction. Most ORFs initiate translation at an ATG start codon except for 6 ORFs, 4 of which have a GTG start codon and the other 2 a TTG start codon. We did not find any tRNA in the genome. Of the 301 predicted ORFs, 110 ORFs (36.4%) had significant homologs in public databases and a biochemical function could be assigned for 62 of these (Table 2, Figure 3). Blast searches revealed that most of the ORFs (66/110) with significant homologs were closely related to other *Myovirida*e (Table 2). Most best-hits corresponded to members of the genus *Phikzvirus*, which comprises myoviruses with a large genome (> 200 kb), including *Pseudomonas* phages Phikz and PhiPA3*, Erwinia* phage *Ea35-70, Ralstonia* phage RSF1 and RSL2 and *Vibrio* phage pVa21, VP4B, pTD1, Phabio, and Noxifer. Although many gene functions could be assigned, most of the predicted genes are ORFans that are unique to BONAISHI.

**Table 2:** Summary table of *Vibrio* phage BONAISHI predicted proteins that contained relevant annotation information as determined from significant BLASTP hits (*e*-value < e-3) against the GenBank non-redundant and CAZY databases.

	**Predicted function**	**START**	**STOP**	**Size (n)**	**Best-hit**	**Classification**	**E-value**	**Score (bits)**	**N°accession**
ORF01c	hypothetical protein	653	1753	1100	*Pseudocercospora musae*	Fungi	2.00E-07	66.00	KNG49823.1
ORF02c	SbcC-like protein	1750	2184	435	*Pseudomonas phage 201phi2-1*	Myoviridae	1.00E-17	88.00	YP_001956973
ORF03	conserved hypothetical protein	2241	3119	879	*Pseudomonas phage Phikz*	Myoviridae	2.00E-32	130.00	NP_803730
ORF05	PaaR repeat-containing protein	3722	4018	297	*Vibrio cholerae*	⋎-proteobacteria	3.00E-21	89.00	WP_057643503
ORF06c	conserved hypothetical protein	4055	4657	603	*Pseudomonas phage Phikz*	Myoviridae	1.00E-25	203.00	AAL83062
ORF07c	virion structural protein	4667	5962	1296	*Pseudomonas phage KTN4*	Myoviridae	7.00E-32	147.00	ANM44952.1
ORF10	ribonuclease H	6889	8271	1383	*Pseudomonas phage 201phi2-1*	Myoviridae	3.00E-25	117.00	YP_001956963
ORF12	*uvsx* protein	9269	10744	1476	*Pseudomonas phage 201phi2-1*	Myoviridae	1.00E-89	290.00	YP_001956960
ORF13	conserved hypothetical protein	10746	11108	363	*Pseudomonas phage phiPA3*	Myoviridae	3.00E-28	108.00	AEH03597
ORF15	virion structural protein	11460	12125	666	*Pseudomonas phage phiPA3*	Myoviridae	2.00E-27	113.00	AEH03595
ORF17	hypothetical protein	12558	13256	699	*Erwinia phage Ea35-70*	Myoviridae	8.00E-09	64.00	YP_009004948
ORF22c	conserved hypothetical protein	16270	18384	2115	*Ralstonia phage RSL2*	Myoviridae	3.00E-74	261.00	BAQ02568
ORF23c	glycoside hydrolase	18393	24110	5718	*Ralstonia phage RP31*	Myoviridae	2.00E-70	277.00	BAW19303.1
ORF24	RNA polymerase beta prime subunit	24183	25850	1668	*Pseudomonas phage OBP*	Myoviridae	1.00E-110	346.00	YP_004958184
ORF25	RNA polymerase beta subunit	25847	28741	2895	*Ralstonia phage RSF1*	Myoviridae	1.00E-102	358.00	BAS04832
ORF26	RNA polymerase beta subunit	29242	30369	1128	*Pseudomonas phage 201phi2-1*	Myoviridae	2.00E-61	223.00	YP_001956996
ORF29	conserved hypothetical protein	32135	32860	726	*Pseudomonas phage Noxifer*	Myoviridae	3.00E-21	110.00	ARV77361.1
ORF30	virion structural protein	32871	33641	771	*Ralstonia phage RSF1*	Myoviridae	3.00E-31	124.00	BAS04828
ORF36c	tail tube protein	36168	37040	873	*Pseudomonas phage 201phi2-1*	Myoviridae	2.00E-24	108.00	YP_001956757
ORF37c	tail shealth protein	37089	39137	2049	*Erwinia phage Ea35-70*	Myoviridae	2.00E-83	286.00	YP_009004971
ORF38	hypothetical protein	39208	40278	1071	*Erwinia phage Ea35-70*	Myoviridae	3.00E-09	67.00	YP_009004972
ORF39	virion structural protein	40288	42819	2532	*Pseudomonas phage 201 phi2-1*	Myoviridae	2.00E-55	226.00	YP_001956754.1
ORF40	virion structural protein	42831	44483	1653	*Pseudomonas phage 201phi2-1*	Myoviridae	3.00E-27	125.00	YP_001956753
ORF41	terminase large subunit	44535	46691	2157	*Pseudomonas phage 201phi2-1*	Myoviridae	1.00E-142	442.00	YP_001956731
ORF43c	conserved hypothetical protein	47798	49027	1230	*Pseudomonas phage Noxifer*	Myoviridae	4.00E-37	164.00	ARV77197.1
ORF44	HD domain protein	49121	49720	600	*Salmonella phage SPN3US*	Myoviridae	3.00E-23	101.00	AEP84084
ORF49c	RNA-binding protein	52606	54225	1620	*Actinomyces massiliensis*	Actinobacteria	2.00E-73	251.00	WP_017194325
ORF58c	conserved hypothetical protein	58317	59075	759	*Erwinia phage Ea35-70*	Myoviridae	1.00E-22	102.00	YP_009005001
ORF59	tubulin-like protein	59178	60167	990	*Erwinia phage Ea35-70*	Myoviridae	3.00E-21	101.00	YP_009005002
ORF68	hypothetical protein	64017	64424	408	*Ralstonia phage RSL2*	Myoviridae	7.00E-06	52.00	BAQ02532
ORF69	conserved hypothetical protein	64596	66782	2187	*Erwinia phage Ea35-70*	Myoviridae	1.00E-105	345.00	YP_009005012
ORF70c	hypothetical protein	66814	67875	1062	*Ralstonia phage RSF1*	Myoviridae	9.00E-08	63.00	BAS05022
ORF71	hypothetical protein	68148	70115	1967	*⋎-protebacteria bacterium*	⋎-proteobacteria	1.00E-15	94.00	OUV32520.1
ORF72	RNA polymerase beta prime subunit	70204	71697	1494	*Erwinia phage Ea35-70*	Myoviridae	3.00E-44	171.00	YP_009005015
ORF73	conserved hypothetical protein	71795	72433	639	*Erwinia phage Ea35-70*	Myoviridae	9.00E-15	79.00	YP_009005021
ORF76	nuclease SbcC subunit	73326	74498	1173	*Pseudomonas phage PhipA3*	Myoviridae	1.00E-46	172.00	AEH03486
ORF77	conserved hypothetical protein	74495	75289	795	*Erwinia phage PhiEaH1*	Siphoviridae	9.00E-23	103.00	YP_009010069
ORF78	hypothetical protein	75301	75972	672	*Uncultured bacterium*	Bacteria	2.00E-06	56.00	EKD22589
ORF79	hypothetical protein	76106	77656	1551	*Pseudomonas phage phiPA3*	Myoviridae	9.00E-06	59.00	AEH03489
ORF80	conserved hypothetical protein	77691	79211	1521	*Pseudomonas phage phiPA3*	Myoviridae	1.00E-16	93.00	AEH03490
ORF81	hypothetical protein	79251	81056	1806	*Gossypium arboreum*	Magnoliopsida	1.00E-06	63.00	KHG21929
ORF82c	hypothetical protein	81109	81456	348	*Pseudomonas phage PA7*	Myoviridae	5.00E-09	59.00	AFO71119
ORF83	RNA polymerase beta subunit	81518	83584	2067	*Pseudomonas phage Phabio*	Myoviridae	8.00E-66	260.00	ARV76743.1
ORF84	RNA polymerase beta prime subunit	83584	85557	1974	*Ralstonia phage RSF1*	Myoviridae	1.00E-50	196.00	BAS05006
ORF85	helicase	85657	87198	1542	*Pseudomonas phage OBP*	Myoviridae	1.00E-40	176.00	AEV89521.1
ORF86	Clp protease subunit	87276	87848	573	*Bacillus cereus*	Bacilli	5.00E-05	52.00	WP_048520069
ORF87	ATP-dependent Clp protease proteolytic subunit	87848	88339	492	*Dactylosporangium aurantiacum*	Actinobacteria	1.00E-27	110.00	WP_033356707
ORF89c	conserved hypothetical protein	88833	90389	1557	*Pseudomonas phage PA7*	Myoviridae	2.00E-15	89.00	AFO71110
ORF92	RNA polymerase beta prime subunit	91656	92915	1260	*Ralstonia phage RSF1*	Myoviridae	8.00E-49	180.00	BAS04991
ORF96c	DNA polymerase *polB*	96090	97820	1731	*Ralstonia phape RP12*	Unclassified virus	6.00E-107	343.00	BAW19225.1
ORF97	virion structural protein	97895	99190	1296	*Erwinia phage PhiEaH1*	Siphoviridae	2.00E-23	111.00	YP_009010288
ORF99c	virion structural protein	101147	104005	2859	*Pseudomonas phage 201phi2-1*	Myoviridae	1.00E-93	327.00	YP_001956873
ORF100c	virion structural protein	104007	105122	1116	*Ralstonia phage RSL2*	Myoviridae	2.00E-49	180.00	BAQ02702
ORF101	capsid protein*	105166	106242	1077	*Ralstonia phage RSF1*	Myoviridae	3.00E-22	105.00	BAS04975
ORF102	virion structural protein	106257	107141	885	*Ralstonia phage RSF1*	Myoviridae	4.00E-07	60.00	BAS04974
ORF104	hypothetical protein	107713	109107	1394	⋎-*protebacteria bacterium*	⋎-proteobacteria	1.00E-12	83.00	OUV32343.1
ORF107	conserved hypothetical protein	111644	113257	1614	*Ralstonia phage RSF1*	Myoviridae	8.00E-22	108.00	BAS04969
ORF108	virion structural protein	113257	114459	1203	*Pseudomonas phage PhiPA3*	Myoviridae	8.00E-12	77.00	AEH03528
ORF110	virion structural protein	115149	116531	1383	*Pseudomonas phage PhiPA3*	Myoviridae	2.00E-28	126.00	AEH03530
ORF111c	helicase	116571	118181	1611	*Pseudomonas phage 201phi2-1*	Myoviridae	2.00E-48	1884.00	YP_001956921
ORF113	major capsid protein	118843	121035	2193	*Erwinia phage Ea35-70*	Myoviridae	2.00E-23	116.00	YP_009005109
ORF115	conserved hypothetical protein	122460	124031	1572	*Ralstonia phage RSL2*	Myoviridae	1.00E-46	179.00	BAQ02643
ORF118c	holliday-junction resolvase	127427	127987	561	*Pseudomonas phage Phabio*	Myoviridae	4.00E-18	99.00	ARV76843.1
ORF119c	virion structural protein	128029	128865	837	*Pseudomonas phage 201phi2-1*	Myoviridae	8.00E-44	159.00	YP_001956947
ORF120c	virion structural protein	128878	130947	2070	*Pseudomonas phage phiPA3*	Myoviridae	7.00E-72	256.00	AEH03570
ORF121	virion structural protein	131049	133649	2601	*Pseudomonas phage phabio*	Myoviridae	4.00E-61	245.00	ARV76832.1
ORF133	hypothetical protein	139060	140100	1041	*Vibrio tasmaniensis*	⋎-proteobacteria	2.00E-11	73.00	WP_017112059
ORF138	glycoside hydrolase	141805	142716	912	*Aureimonas altamirensis*	α-proteobacteria	2.00E-49	174.00	BAT26087
ORF141	hypothetical protein	145070	148201	3132	*Psychromonas ingrahamii*	⋎-proteobacteria	1.00E-08	72.00	WP_011768462.1
ORF143	hypothetical protein	150013	151644	1631	*Vibrio phage s4-7*	Unclassified virus	2.00E-06	63.00	AOQ26845.1
ORF151	hypothetical protein	160192	161433	1242	*Colwellia phage 9A*	Siphoviridae	4.00E-08	66.00	YP_006489231
ORF159	hypothetical protein	168857	169801	945	*Escherichia phage phAPEC8*	Myoviridae	4.00E-10	68.00	YP_007348452
ORF163	hypothetical protein	172164	172847	684	*Ruegeria halocynthiae*	α-proteobactérie	1.00E-19	91.00	WP_037312174
ORF167	dead-like helicase	176707	178806	2100	*Erwinia phage Ea35-70*	Myoviridae	1.00E-114	367.00	YP_009004923
ORF172	nicotinamide-nucleotide adenylyltransferase	182827	184476	1650	*Vibrio phage 11895-B1*	Myoviridae	1.00E-106	331.00	YP_007673553
ORF176	nicotinamide-nucleotide adenylyltransferase	185996	187114	1119	*Thiorhodococcus drewsii*	⋎-proteobacteria	3.00E-46	170.00	WP_007039048
ORF177	nicotinamide phosphoribosyltransferase	187169	188668	1500	*Vibrio nigripulchritudo*	⋎-proteobacteria	3.00E-59	210.00	WP_022562194
ORF181	nicotinamide riboside transporter	190786	191502	717	*Vibrio phage 11895-B1*	Myoviridae	3.00E-76	238.00	YP_007673552
ORF189	hypothetical protein	196049	196543	717	*Kaistia granuli*	α-proteobacteria	1.00E-08	57.00	WP_018183972
ORF198	NAD-dependent DNA ligase	204242	206257	2016	*Vibrio maritimus*	⋎-proteobacteria	0.00E+00	608.00	WP_042496716
ORF200	phosphatase	206698	207330	633	*Vibrio phage VH7D*	Myoviridae	6.00E-19	89.00	YP_009006310
ORF206	hypothetical protein	210803	211318	516	*Psychromonas aquimarina*	⋎-proteobacteria	7.00E-34	126.00	WP_028862581
ORF209	hypothetical protein	213895	214209	315	*Enterovibrio calviensis*	⋎-proteobacteria	6.00E-07	52.00	WP_017007757
ORF212	transcriptional regulator	215240	215764	525	*Leptolyngbya sp. PCC 7375*	cyanobacteria	1.00E-05	53.00	EKV01169
ORF220	hypothetical protein	220875	221303	429	*Shewanella sp. phage 1/4*	Myoviridae	6.00E-11	64.00	YP_009100318
ORF227	hypothetical protein	224066	224998	933	*Pseudomonas phage PhiPA3*	Myoviridae	9.00E-06	57.00	AEH03433
ORF229	hypothetical protein	226086	227921	1835	*Vibrio phage RYC*	Unclassified virus	7.00E-70	243.00	BAV81012.1
ORF231	thymidylate kinase	228796	229458	663	*Desulfosporosinus acidiphilus*	Clostridia	8.00E-42	150.00	WP_014828008
ORF232	ribonucleotide-diphosphate reductase subunit alpha	229535	230446	912	*Aeromonas molluscorum 848*	⋎-proteobacteria	1.00E-132	385.00	EOD53957
ORF233	ribonucleotide-diphosphate reductase subunit alpha	230844	232277	1434	*Neisseria meningitidis*	β-proteobacteria	0.00E+00	635.00	WP_049227356
ORF234	ribonucleotide-diphosphate reductase subunit beta	232355	233062	708	*Thiomicrospira sp. Kp2*	⋎-proteobacteria	3.00E-96	294.00	WP_040727751
ORF235	HNH endonuclease	233205	233927	723	*Bacillus pumilus*	Bacilli	7.00E-32	123.00	WP_051149989
ORF236	ribonucleotide-diphosphate reductase subunit beta	234231	234617	387	*Shigella sonnei*	⋎-proteobacteria	2.00E-36	129.00	CSE34793
ORF238	hypothetical protein	235508	237232	1725	*Polyangium brachysporum*	β-proteobacteria	2.00E-23	114.00	WP_047195109
ORF249c	hypothetical protein	242652	244886	2235	*Vibrio mimicus*	⋎-proteobacteria	4.00E-08	67.00	WP_001015571
ORF253	phosphatase	246359	247015	657	*Verrucomicrobium spinosum*	verucomicrobia	2.00E-22	97.00	WP_009962909
ORF254	hypothetical protein	247012	247842	831	*Shewanella sp*.	⋎-proteobacteria	6.00E-06	57.00	WP101034114.1
ORF255	ATP-binding protein	247870	248553	684	*Vibrio phage RYC*	Unclassified virus	1.00E-36	137.00	BAV81012.1
ORF256c	T5 A1-like protein	248608	250479	1872	*Caulobacter phage phiCbK*	Siphoviridae	3.00E-83	281.00	YP_006988022
ORF257c	hypothetical protein	250484	250927	444	*Pseudoalteromonas (multispecies)*	⋎-proteobacteria	9.00E-19	86.00	WP_024591352
ORF258	conserved hypothetical protein	251087	252592	1506	*Campylobacter phage CP30A*	Myoviridae	3.00E-20	102.00	YP_006908082
ORF259	conserved hypothetical protein	252655	253968	1314	*Campylobacter phage CP30A*	Myoviridae	2.00E-18	96.00	YP_006908082
ORF261	phosphate starvation protein PhoH	254632	255627	996	*Corynebacterium glucuronolyticum*	Actinobacteria	6.00E-49	175.00	WP_005389286
ORF269	thymidylate synthase-complementing protein	260653	262095	1443	*Parcubacteria*	Parcubacteria	5.00E-62	217.00	KKR42866
ORF272	conserved hypothetical protein	263205	263723	519	*Cronobacter phage vB_CsaM_GAP32*	Myoviridae	9.00E-15	77.00	YP_006987447
ORF289	conserved hypothetical protein	273177	273917	740	*Vibrio phage vB_VhaS-a*	Unclassified virus	4.00E-18	100.00	ANO57550.1
ORF290	conserved hypothetical protein	273997	274665	668	*Vibrio phage vB_VhaS-a*	Unclassified virus	1.00E-14	88.00	ANO57549.1
ORF295c	hypothetical protein	276639	277370	731	*Ralstonia phage RP12*	Myoviridae	9.00E-10	73.00	BAW19047.1
ORF300	hypothetical tail protein	286648	287631	983	*Pseudomonas phage Phabio*	Myoviridae	7.00E-15	90.00	ARV76834.1
ORF301	virion structural protein	287726	288967	1241	*Pseudomonas phage Noxifer*	Myoviridae	3.00E-07	65.00	ARV77324.1

### Gene annotation

#### Proteins involved in virion structure and assembly

**Figure 4:**
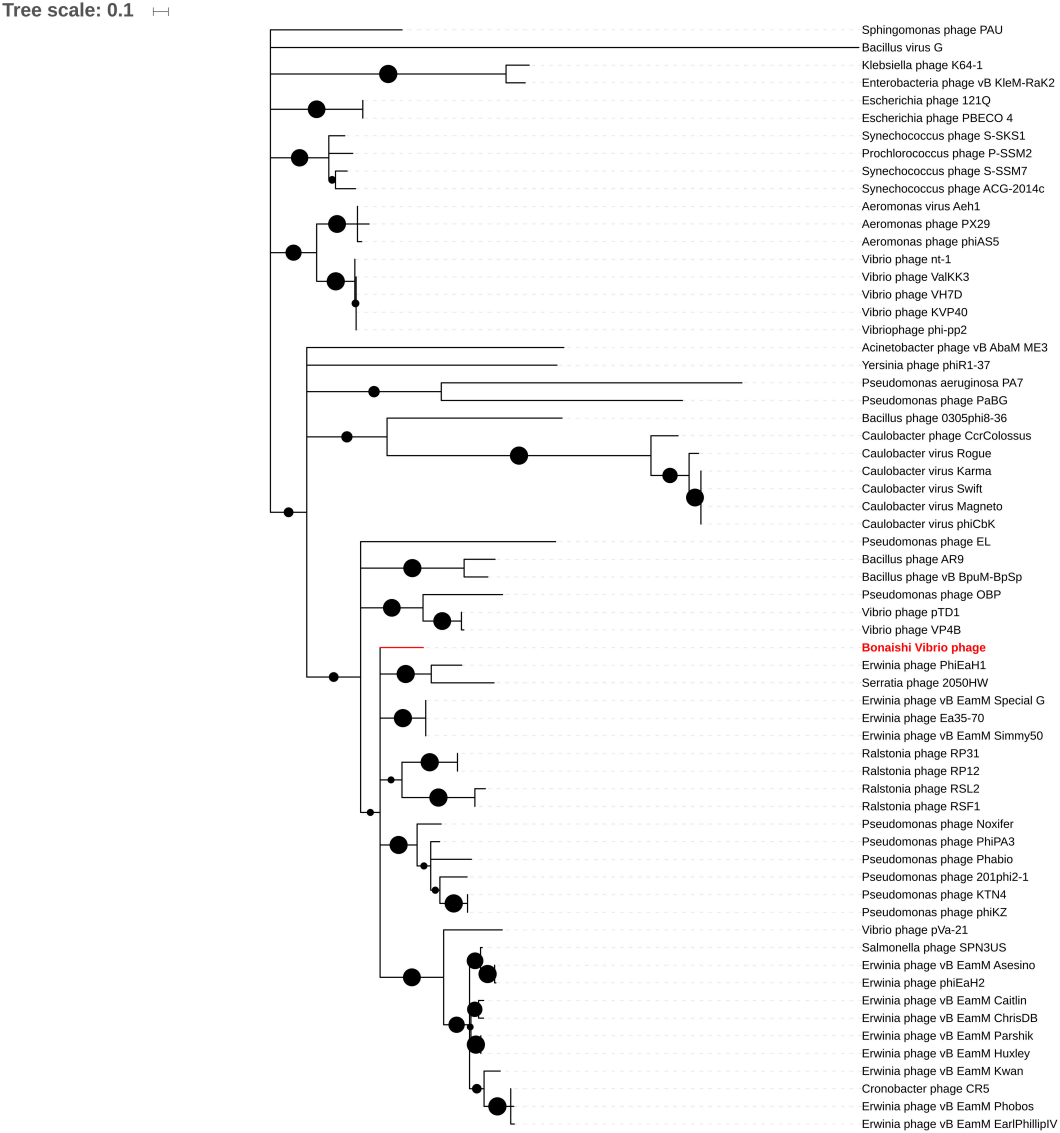
Phylogenetic analysis of 63 Jumbo phages based on the amino acids sequence of the terminase large subunit. Sequences were aligned by Muscle and the tree was constructed by Maximum Likelihood method with a bootstrap of 1,000 using Mega 6.0 (Tamura et al., 2013) as in Yuan and Gao (2017). Bootstrap values higher than 0.5 are represented by black circles, ranging from 0.5 (smaller circle) to 1 (bigger circle).

The predicted proteins involved in the virion structure and assembly included a major and an accessory capsid protein (ORF 101 and 113), several tail components including a tail tube (ORF 36), a tail shealth (ORF 37), and an accessory tail protein (ORF 300), as well as several conserved structural proteins (ORFs 7, 15, 30, 39, 40, 97, 99, 100, 102, 108, 110, 119, 120, 121, and 301). We also identified a terminase large subunit (ORF 41) involved in DNA packaging. All these structural components shared strong homologies with proteins encoded by other members of *Phikzvirus* genus (Table 1, Figure S1). Phylogenetic analyses based on the amino acid sequence of the terminase large subunit classified BONAISHI as a singleton (Figure 4). The closest sequences belong to *Vibrio* phage pVa-21 and a cluster with *Cronobacter* phage CR5, *Erwinia* phage vB EamM, phiEaH2 and *Salmonella* phage SPN3US. The second closest cluster comprised *Phikzvirus Pseudomonas* phage KTN4, Phikz, Noxifer, PA3, Phabio, and 201 phi2-1.

#### Proteins involved in dna replication, recombination and repair

The BONAISHI genome encoded at least 10 proteins involved in DNA replication, recombination, and repair. These included a DNA polymerase B (ORF 96), putative DnaB, and DEAD-like helicases (ORF 85 and 111), NAD-dependent DNA ligase (ORF 198), SbcC-like nucleases (ORF2 and 76), a ribonuclease H (ORF 10), Holliday junction resolvase (ORF 118), uvsx recombinase (ORF12). ORF 235 corresponded to the HNH family of homing endonuclease located between genes encoding β subunits of ribonucleotide diphosphate reductase.

#### Proteins involved in nucleotide metabolism and dna modification

We were able to assign a putative function to 10 enzymes involved into nucleotide metabolism. Predicted proteins included two ribonucleotide diphosphate reductase (RDR) α subunits (ORFs 232 and 233) and two RDR β subunits (ORFs 234 and 236), 4 proteins of the pyridine nucleotide salvage pathway corresponding to a nicotinamide riboside transporter (ORF 181), a nicotinamide phosphoribosyltransferase (ORF 177) as well as nicotinamide mononucleotide adenylyltransferases (ORF 172 and 176). BONAISHI also encodes 2 proteins involved in thymidine biosynthesis one of which is a thymidylate synthase complementing protein (ORF 269) and the other a thymidylate kinase (ORF 231). We did not identify any gene for protein involved in DNA modification.

#### Proteins involved in dna transcription

A transcription regulator related to the PadR family (ORF 212) was found as well as two sets of multisubunit RNA polymerase genes (β- and β'- RNAP subunits). ORFs 24, 25, 26, 92 corresponded to the Phikz virion-associated RNAP and ORFs 72, 83, 84 shared significant homologies with the Phikz genes encoding the early expressed RNAP (Table 2, Figure S1). No homologs of the early expressed Phikz β- RNAP subunits were found in BONAISHI genome. ORF 49 was assigned to an RNA binding protein.

#### Lysis, host-phage interaction, and lysogeny

We were able to annotate two proteins (ORFs 23 and 138) involved into host-virus interactions. There were good blastp hits on proteins of unknown function in the NCBI nr database that could be expanded to known glycoside hydrolases (GH) in the expert database CAZY (Carbohydrate Active enZYmes db, http://www.cazy.org). These included a glycoside hydrolase with a conserved endopeptidase domain (ORF 23) affiliated to the GH23 family, which mostly include lysozymes. The protein encoded by ORF 138 belongs to the glycoside hydrolase family GH19, which comprises chitinases and lysozymes. Both enzyme groups catalyze the hydrolysis of polysaccharides containing N-Acetylglucosamine, but the gene sequence does not discriminate between the two. No genes implicated in lysogeny establishment or control (integrase/excisionase, transposases, ParA/ParB genes, attachment sites, transcription repressor, etc.) were detected in the BONAISHI genome, even using prophage expert databases, which confirms that it is exclusively lytic. In addition, blastp searches using BONAISHI hosts genome did not find evidence of the presence of BONAISHI as prophage. Only three genes involved in DNA replication and recombination (ORF 225), nucleotide metabolism (ORF 192) and gene encoding for a phosphate starvation protein showed high score but the similarity was relatively low (<77%).

#### Miscellaneous proteins

We identified a phosphate starvation protein PhoH (ORF 261). This protein has been reported in many other *Myoviridae*. For therapeutic applications of this phage, we also looked for potential toxin encoding genes using BLASTP on 4 toxin expert databases. No homologs to currently known toxins were detected.

#### Metagenomic analysis

The global distribution of BONAISHI was investigated using marine viromes from various waters including coral mucus and the water from coral reefs (Table S2). The highest number of recruited reads was from the virome collected at ALOHA station in the North Pacific subtropical gyre (CAM_SMPL_00823): BONAISHI recruited 1.04% of the reads with a mean identity of 58.23%. None of the recruited reads exceeded 85% identity. We also used prokaryotic metagenomes to test whether the BONAISHI genome is found as a prophage in bacterial hosts. No bacterial reads were recruited.

### Preliminary therapy assays of diseased symbiodinium

**Figure 5:**
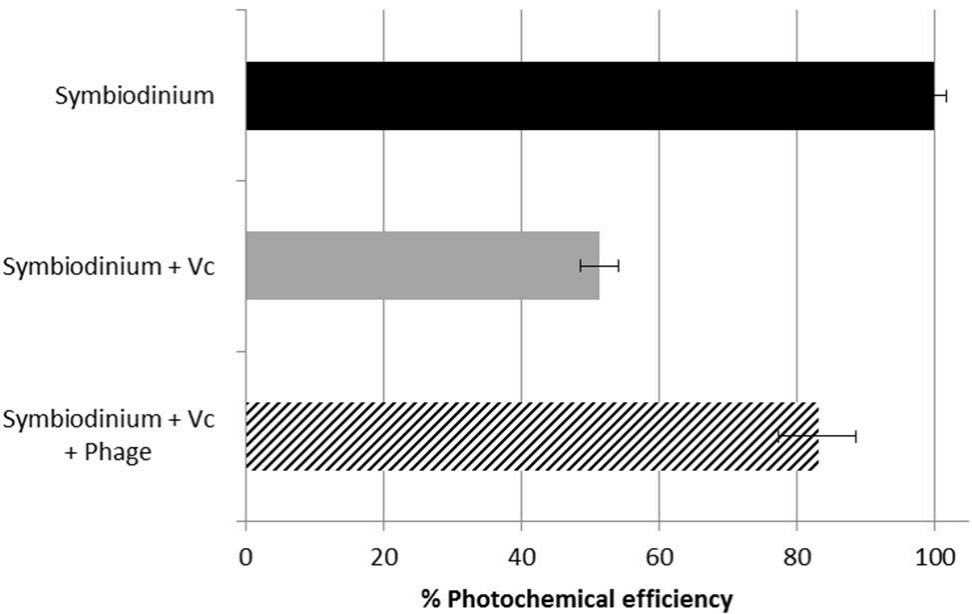
Quantum yield (Fv/Fm) of the photosystem II of control *Symbiodinium* sp. culture (black), *Symbiodinium* sp. inoculated with *V. coralliilyticus* YB1 pathogen (gray), and *Symbiodinium* sp. co-inoculated with *V. coralliilyticus* YB1 pathogen and *Vibrio* phage BONAISHI (hashed) after 60 min incubation. Results are expressed as the % of Fv/Fm in the control culture. As reported in previous study, the inoculation of *V. coralliilyticus* YB1 pathogen induced a rapid decline in *Symbiodinium* sp. photochemical efficiency. The addition of BONAISHI rapidly counteracted the impact of *V. coralliilyticus* YB1 on the efficiency of *Symbiodinium* photochemical activity.

*Symbiodinium* sp. control culture showed optimal photosynthetic activity with the quantum yield Fv/Fm between 0.57 and 0.59 during the course of the experiment. As expected, inoculation with *V. coralliilyticus* caused a rapid photoinhibition with a 50% decrease in the quantum yield 60 min after inoculation (Figure 5). BONAISHI was able to significantly counteract the bacterial algicidal activity (*t*-test, *p* < 0.001) as, with both *V. coralliilyticus* and BONAISHI, the quantum yield was only 14% lower than the control 60 min after inoculation.

## Discussion

In recent years, *Vibrio coralliilyticus* has been used as a model pathogen to gain insights into the establishement and propagation of coral diseases (Sussman et al., 2008; O'Santos et al., 2011; Garren et al., 2014; Pollock et al., 2015). The use of phages to control *V. coralliilyticus* has been reported recently (Efrony et al., 2007; Cohen et al., 2013) but if phage therapy is to become a practical approach, fundamental knowledge on pathogen-virus interactions must be investigated in detail to evaluate, on the one hand, the therapeutic potential of the candidate phage and, on the other hand, the suitability of the host for phage therapy.

The detection of virus-derived genes in the *Vibrio coralliilyticus* P1 and YB1 genomes (Weynberg et al., 2015) shows that these pathogenic strains have interacted with phages during the course of their evolutionary history. Past interactions events with viruses can lead to the development of resistance mechanisms to escape phage infection, which may, in turn, limit the application of phage therapy. We, however, did not detect any of the distinctive genetic signatures of viral resistance mechanisms, such as the insertion of short palindromic sequences (CRISPR, data not shown) in *V. coralliilyticus* P1 and YB1. Although other resistance mechanisms exist, the recurrent isolation of phages that infect *V. coralliilyticus* YB1 and/or P1 (Efrony et al., 2007; Cohen et al., 2013; Ramphul et al., 2017) supports the idea that *V. coralliilyticus* pathogens are permissive to viral infection and are suitable candidates for treatment by phage therapy.

The *Vibrio* phage BONAISHI isolated from coastal waters in the South China Sea is distinct from the known *V. coralliiltyicus* phages YB2, YC, CKB-S1, CKB-S2, RYC (Efrony et al., 2007; Cohen et al., 2013; Ramphul et al., 2017). Although all these phages belong to the order of *Caudovirales* (tailed bacteriophages), BONAISHI has an unusually large genome, 303 kbp, rather than 11 kbp to 158 kbp. With such a large genome, BONAISHI is a novel jumbo phage (or giant phage). These are tailed phages with a dsDNA genome larger than 200 kb (Hendrix, 2009; Yuan and Gao, 2017). Jumbo phages have often been isolated in recent years and they mostly infect Gram-negative bacteria, including the genera *Synechoccocus, Vibrio, Pseudomonas, Caulobacter, Erwinia*, and *Aeromonas* (Yuan and Gao, 2017). As observed for most jumbo phages, the large genome of BONAISHI is packaged in a large head connected to a long, contractile tail. The genome of jumbo phages typically includes core genes involved in virion structure and assembly, DNA replication, and nucleotide metabolism, including several genes encoding multisubunit RNAP. In BONAISHI, the core genes are scattered throughout the genome sequence and most of them share significant homology with other jumbo phages affiliated to the *Phikzvirus* genus. BONAISHI is the first marine representative of this divergent group within the *Myoviridae*. Interestingly, many Phikzviruses are considered to be promising biocontrol agents for plant-pathogenic bacteria (*Ralstonia solanacearum, Erwinia amylovora*) and some of them are already found in commercial products for phage therapy (Fujiwara et al., 2011; Bhunchoth et al., 2016).

The life history traits and genomic analysis showed that *Vibrio* phage BONAISHI is a good candidate for biological control of *V. coralliilyticus*. Firstly, BONAISHI appears to be structurally stable as it can withstand a wider range of pH (3–10) and temperature (4–45°C) than in the environment where it would be used. Second, it readily infects and lyses several pathogenic strains of *V. coralliilyticus* but no related species. Thirdly incubation experiments showed that the replication cycle is fast (latent period < 3 h). The presence of genes encoding virion-associated RNAP (ORFs 24, 25, 26, and 92) and early-expressed RNAP (ORFs 72, 83, and 84) in the BONAISHI genome may, at least partly, explain the rapid cycle (Ceyssens et al., 2014). In Phikzviruses, these two sets of RNAP may operate in concert during the replication cycle (Ceyssens et al., 2014; Yuan and Gao, 2017). The virion-associated RNAP may be injected into the host cell to start immediate gene expression whereas the early expressed RNAP may function during the middle and late phases of phage gene expression. The consecutive action of these enzymes, unique to Phikzviruses, confers the ability to produce viral progeny independently of the host transcription apparatus (Ceyssens et al., 2014; Yuan and Gao, 2017). In addition, the absence of detectable tRNA in BONAISHI genome suggests that it is well adapted to the translation machinery of its hosts, which is a critical process for efficient phage propagation. Finally, the genomic analysis did not identify any temperate phage hallmarks such as integration mediating enzymes, or genome architecture or sequence similarity with known temperate phages. Furthermore, the absence of homology between BONAISHI gene sequences and bacteria reads from coral metagenomes supports the idea that this phage does not integrate into the host genome. These results suggest very strongly that the *Vibrio* phage BONAISHI is a strictly lytic phage that is species specific and stable, although it appears to be relatively rare in the environment.

Another important issue for therapeutic applications of phages is to ensure that the candidate does not perform specialized or generalized transduction (Duckworth and Gulig, 2002). Given that BONAISHI appears to be strictly lytic based on the growth experiments and the genome analysis, it is unlikely that this candidate will perform specialized transduction of host DNA. Specialized transduction is restricted to temperate phages and occurs when the prophage is not cleanly excised during induction and includes the flanking bacterial genes which are then packaged in the viral progeny. Our candidate, however, may be able to perform generalized transduction. In this type of transduction, random segments of degraded host chromosome are mistakenly packaged instead of the phage DNA and may be transmitted by horizontal gene transfer. Phages that use a headful DNA packaging mechanism, such as many jumbo phages including BONAISHI, may be able to perform generalized transduction. However, it is, to the best of our knowledge, impossible to predict the frequency of generalized transduction based purely on the genome analysis. For example, giant bacteriophages with similar headful DNA packaging mechanisms can have very different transduction rates as, for example, the T4 and T4G bacteriophages (Young et al., 1982; Young and Edlin, 1983). The ability of BONAISHI to perform generalized transduction would, therefore, require proper laboratory investigation.

An alternative to avoid potential issue with phage-mediated gene transfer is, rather than using whole bacetriophages, to use bacteriolytic proteins encoded by phages, among which the most notable are phage-encoded peptidoglycan hydrolases (PGH, see review by Roach and Debarbieux, 2017). PGHs, also called endolysins, degrade the cell peptidoglycan from within and contribute to the release of progeny and cell burst. A second type of PGH can be associated with the virion and initiate cell wall penetration through localized peptidoglycan or lipopolysaccharide degradation during the infection process. Both types of PGH are already used as bacteriocins in animal models of human infection and disease (see Roach and Debarbieux, 2017 and references therein). Jumbo phages typically encode more proteins for the lysis of the host cell wall including endolysin, glycoside hydrolase and chitinase, which are often bound to the virion than small genome phages (Yuan and Gao, 2017). In BONAISHI genome, we identified two glycoside hydrolases distantly related to known enzymes that belong to the families GH19 and GH23 using the expert database CAZY. Although the catalytic activities of these molecules cannot be determined based solely on the genome analysis, their overexpression and characterization might provide interesting tools for controlling *V. coralliilyticus* infection.

A preliminary assay suggests that BONAISHI is a promising candidate for treating *V. coralliilyticus* infection. Studies investigating the action of *V. coralliilyticus* on coral symbionts showed that photosynthesis was inactivated by the expression of a Zn-metalloprotease (Sussman et al., 2009). Our experiments on *Symbiodinum* cultures infected by *V. coralliilyticus* showed that BONAISHI phage treatment was effective: adding the phage to the infected cultures rapidly reduced *Symbiodinium* PSII inactivation. As reported in previous studies, phage addition probably lysed the bacterial pathogen, stopping Zn-metalloprotease production and further damage to *Symbiodinium* sp. cells (Cohen et al., 2013). Future studies should now focus on the effectiveness of the treatment either under realistic field conditions or in mesocosms to start including bacteriophages (and/or derived compounds) in an integrated management program to mitigate the damage caused by the infectious agents responsible for coral diseases. We recommend genome sequencing and analysis of any future phage candidate as a prerequisite to any field test to ensure safe environmental applications as this provides essential information on the phage replication cycle and host-virus interactions.

## Author contributions

A-CB, YB, and TB, designed the study. LJ, JM, and A-CB performed the experiments and analyzed the results. LJ, SH, CD, and EC performed the bioinformatics analyses. CF-P provided and helped with the diseased *Symbiodium* cultures. LJ and A-CB wrote the manuscript.

### Conflict of interest statement

The authors declare that the research was conducted in the absence of any commercial or financial relationships that could be construed as a potential conflict of interest.
