# Supplementary material for: Therapeutic Potential of a New Jumbo Phage That Infects Vibrio coralliilyticus, a Widespread Coral Pathogen
Source: Front Microbiol. 2018 Oct 24;9:2501. doi: 10.3389/fmicb.2018.02501 (PMC6207643; doi:10.3389/fmicb.2018.02501)

**Figure S1:** Similarity of annotated functional genes in BONAISHI with related jumbo phages. Nucleic acid query sequences in BONAISHI were compared with the complete genes sequences of related phages by TBLASTX. We conserved hits with an evalue smaller than 0.001 and an alignment lenght higher than 50 bp. Green color intensity represent similarity percentage among homologs.


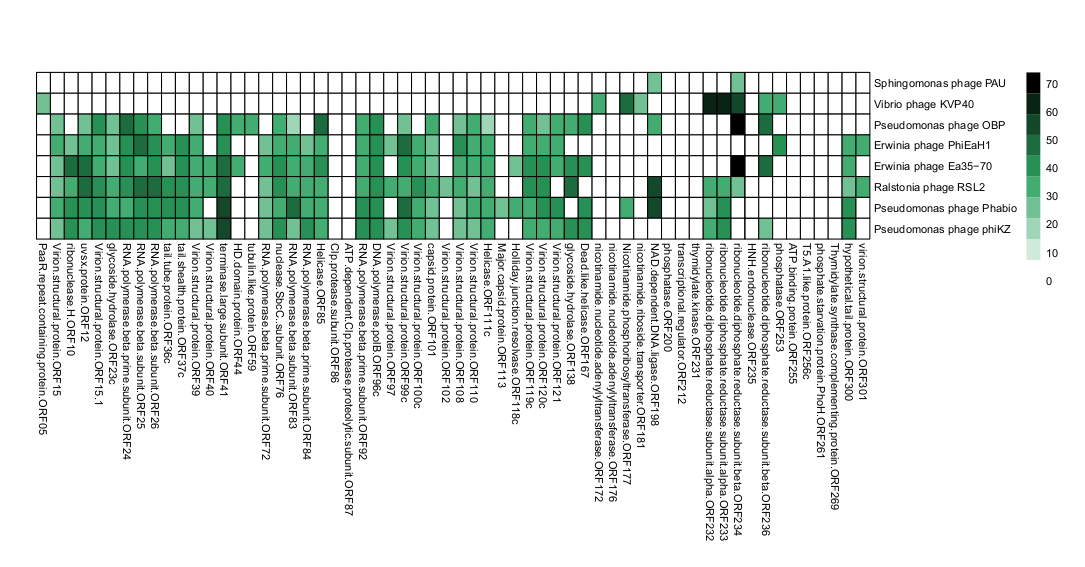

Supplement: Supplementary file 1 [file Data_Sheet_1.docx]
