# Supplementary material for: Therapeutic Potential of a New Jumbo Phage That Infects Vibrio coralliilyticus, a Widespread Coral Pathogen
Source: Front Microbiol. 2018 Oct 24;9:2501. doi: 10.3389/fmicb.2018.02501 (PMC6207643; doi:10.3389/fmicb.2018.02501)
Supplement: Supplementary file 2 [file Data_Sheet_2.docx]

Table S1: List of bacteria tested for the determination of the host range of *Vibrio phage* BONAISHI. For the pairwise infections, a 5 µL aliquot of freshly produced BONAISHI lysate was spotted on a lawn of exponentially growing bacteria. The formation of plaques was recorded after 24 and 48h. ⦁ indicates lysis.

|  | ***V. corallilyticus* phage BONAISHI** |
| --- | --- |
| *Photobacterium rosenbergii* LMG22226 ^a^ |  |
| *Vibrio shiloni* LMG19703 ^a^ |  |
| *Enterovibrio coralli* LMG22228 ^a^ |  |
| *Vibrio coralliilyticus* LMG21348 ^a^ | • |
| *Vibrio coralliilyticus* LMG20984 ^a^ | • |
| *Vibrio coralliilyticus* LMG23696 ^a^ | • |
| *Vibrio sp. cf coralliilyticus* 1H13 ^b^ | • |
| *Vibrio sp. cf coralliilyticus* WH2 ^b^ |  |
| *Vibrio tubiashi* LMG10936T ^a^ |  |
| *Vibrio alginolyticus* 9H16 ^b^ |  |
| *Vibrio alginolyticus* 8B ^b^ |  |
| *Vibrio alginolyticus* 44097 ^c^ |  |
| *Vibrio sp. cf alginolyticus* 9H3 ^b^ |  |
| *Vibrio sp. cf alginolyticus* 3B32 ^b^ |  |
| *Vibrio sp. cf alginolyticus* 1H9 ^b^ |  |
| *Vibrio sp. cf alginolyticus* 3B21 ^b^ |  |
| *Vibrio sp. cf alginolyticus* 8B9 ^b^ |  |
| *Vibrio parahaemolyticus* LMG2850 ^a^ |  |
| *Vibrio parahaemolyticus* LMG4423 ^a^ |  |
| *Vibrio sp. cf parahaemolyticus* 4B25 ^b^ |  |
| *Vibrio harveyi* LMG19714 ^a^ |  |
| *Vibrio harveyi* ORM4 ^c^ |  |
| *Vibrio harveyi* LMG4044 ^a^ |  |
| *Vibrio harveyi* 7B6 ^b^ |  |
| *Vibrio harveyi* 8B6 ^b^ |  |
| *Vibrio sp. cf harveyi* 5B8 ^b^ |  |
| *Vibrio sp cf. harveyi* SWAT3 ^d^ |  |
| *Vibrio sp. communis* WB3 ^b^ |  |
| *Vibrio nigripulchritudo* 5065 ^c^ |  |
| *Vibrio nigripulchritudo* SFn1 ^c^ |  |
| *Vibrio campbelli* LMG11216 ^a^ |  |
| *Vibrio aesturianus* 02/041 ^c^ |  |
| *Vibrio vulnificus* CIP 755AT ^c^ |  |
| *Vibrio tapetis* LP2 ^c^ |  |
| *Vibrio tapetis* CECT4600 ^c^ |  |
| *Vibrio splendidus* MEL32 ^c^ |  |
| *Vibrio splendidus* ATCC25914 ^e^ |  |
| *Vibrio sp.*1H11 ^b^ |  |
| *Vibrio sp.* 8B12 ^b^ |  |
| *Vibrio sp.* 9H4 ^b^ |  |
| *Vibrio sp.* 8B23 ^b^ |  |
| *Vibrio sp.* cf gallicus 4B2 ^b^ |  |
| *Vibrio sp.* cf gallicus/ xuii 4B3 ^b^ |  |
| *Listonella anguillarum* 4B1 ^b^ |  |

^a^ BCCM/ LMG Bacteria Collection, ^b^ Baudoux private bacteria collection (Station Biologique de Roscoff), ^c^ Paillard private bacteria collection (Institut Universitaire Européen de la Mer), ^d^ Azam private bacteria collection (Scripps Institute of Oceanography), ^e^ ATCC Bacteriology Collection
