# Supplementary material for: Therapeutic Potential of a New Jumbo Phage That Infects Vibrio coralliilyticus, a Widespread Coral Pathogen
Source: Front Microbiol. 2018 Oct 24;9:2501. doi: 10.3389/fmicb.2018.02501 (PMC6207643; doi:10.3389/fmicb.2018.02501)
Supplement: Supplementary file 3 [file Data_Sheet_3.docx]

| **Sample ID** | **reads number** | **Database** | **Sample name** | **Environment** | **nbr hits >=50% length** | **% recruited reads (>=50% length)** |
| --- | --- | --- | --- | --- | --- | --- |
| CAM_SMPL_000713 | 244067 | Broadphages-iMicrobes | Uncultured virome Virome 4 | gut | 83 | 0.03 |
| CAM_SMPL_000717 | 213171 | Broadphages-iMicrobes | Virome Kelp forest virome | seawater | 0 | 0.00 |
| CAM_SMPL_000719 | 127601 | Broadphages-iMicrobes | Virome EPR hydrothermal vent: Extracellular ssDNA virome | seawater | 2 | 0.00 |
| CAM_SMPL_000720 | 123595 | Broadphages-iMicrobes | Virome EPR hydrothermal vent: Induced RNA virome | seawater | 4 | 0.00 |
| CAM_SMPL_000722 | 172745 | Broadphages-iMicrobes | Virome FeCl method-1 | seawater | 98 | 0.06 |
| CAM_SMPL_000723 | 94832 | Broadphages-iMicrobes | Virome FeCl method-2 | seawater | 64 | 0.07 |
| CAM_SMPL_000724 | 175121 | Broadphages-iMicrobes | Virome FeCl method-3 | seawater | 95 | 0.05 |
| CAM_SMPL_000725 | 218300 | Broadphages-iMicrobes | Virome FeCl method-4 | seawater | 99 | 0.05 |
| CAM_SMPL_000726 | 162078 | Broadphages-iMicrobes | Virome FeCl method-5 | seawater | 95 | 0.06 |
| CAM_SMPL_000727 | 124120 | Broadphages-iMicrobes | Virome FeCl method-6 | seawater | 72 | 0.06 |
| CAM_SMPL_000799 | 205938 | Broadphages-iMicrobes | Black Sea Sediment Metagenome | sediment | 0 | 0.00 |
| CAM_SMPL_000800 | 246799 | Broadphages-iMicrobes | Virome Lake227 Freshwater Metagenome | freshwater | 0 | 0.00 |
| CAM_SMPL_000801 | 229981 | Broadphages-iMicrobes | Virome_HOT219S2C13_F2 | seawater | 209 | 0.09 |
| CAM_SMPL_000803 | 110191 | Broadphages-iMicrobes | Virome Anaerobic oil degrading sediment virome | marine sediment | 0 | 0.00 |
| CAM_SMPL_000804 | 123131 | Broadphages-iMicrobes | Virome Guaymas hydrothermal vent: Extracellular ssDNA virome | seawater | 3 | 0.00 |
| CAM_SMPL_000805 | 239275 | Broadphages-iMicrobes | Virome Archaeal dominated cold seeps Costa Rica RNA virome | seawater | 2 | 0.00 |
| CAM_SMPL_000806 | 202505 | Broadphages-iMicrobes | Virome Tampa Bay lytic virome | seawater | 8 | 0.00 |
| CAM_SMPL_000807 | 204594 | Broadphages-iMicrobes | 12C Fraction ANME Virome | sediment | 0 | 0.00 |
| CAM_SMPL_000808 | 132133 | Broadphages-iMicrobes | Virome ME-08-9 | seawater | 2 | 0.00 |
| CAM_SMPL_000809 | 89377 | Broadphages-iMicrobes | Virome Guaymas hydrothermal vent: Induced RNA virome | seawater | 1 | 0.00 |
| CAM_SMPL_000811 | 227899 | Broadphages-iMicrobes | Virome Arctic cryopeg brine | seawater | 10 | 0.00 |
| CAM_SMPL_000812 | 227662 | Broadphages-iMicrobes | Virome Arctic frost flowers | seawater | 15 | 0.01 |
| CAM_SMPL_000813 | 214449 | Broadphages-iMicrobes | 1000 meters DNA | seawater | 1 | 0.00 |
| CAM_SMPL_000814 | 200505 | Broadphages-iMicrobes | Virome Lake239 Freshwater Metagenome | freshwater | 0 | 0.00 |
| CAM_SMPL_000815 | 219175 | Broadphages-iMicrobes | Kaneohe Bay RNA metagenome | seawater | 0 | 0.00 |
| CAM_SMPL_000816 | 273303 | Broadphages-iMicrobes | Virome_HOT219S2C13_F4 | seawater | 8 | 0.00 |
| CAM_SMPL_000817 | 199514 | Broadphages-iMicrobes | 13C-Enriched Aurobic Sediment Virome | sediment | 0 | 0.00 |
| CAM_SMPL_000818 | 206759 | Broadphages-iMicrobes | Virome ANME virome | marine sediment | 5 | 0.00 |
| CAM_SMPL_000819 | 150076 | Broadphages-iMicrobes | Virome ME-08-1 | seawater | 10 | 0.01 |
| CAM_SMPL_000820 | 170860 | Broadphages-iMicrobes | Virome ME-08-4 | seawater | 395 | 0.23 |
| CAM_SMPL_000821 | 169279 | Broadphages-iMicrobes | Virome ME-08-8 | seawater | 5 | 0.00 |
| CAM_SMPL_000822 | 169419 | Broadphages-iMicrobes | Virome Guaymas hydrothermal vent: Induced ssDNA virome | seawater | 1 | 0.00 |
| CAM_SMPL_000823 | 225223 | Broadphages-iMicrobes | Virome_HOT219S2C13_F1 | seawater | 2343 | 1.04 |
| CAM_SMPL_000824 | 205875 | Broadphages-iMicrobes | Kaneohe Bay metagenome | seawater | 1 | 0.00 |
| CAM_SMPL_000825 | 90174 | Broadphages-iMicrobes | Virome Archimedes3 | seawater | 26 | 0.03 |
| CAM_SMPL_000826 | 166940 | Broadphages-iMicrobes | Virome ME-08-2 | seawater | 97 | 0.06 |
| CAM_SMPL_000827 | 136262 | Broadphages-iMicrobes | Virome ME-08-6 | seawater | 25 | 0.02 |
| CAM_SMPL_000828 | 45494 | Broadphages-iMicrobes | Virome ME-08-7 | seawater | 6 | 0.01 |
| CAM_SMPL_000829 | 239048 | Broadphages-iMicrobes | 1000 meters RNA | seawater | 3 | 0.00 |
| CAM_SMPL_000830 | 189076 | Broadphages-iMicrobes | Virome BSL DNA virus 2 virome | hot springs | 0 | 0.00 |
| CAM_SMPL_000831 | 228827 | Broadphages-iMicrobes | Virome Benthic methanotrophic mats virome | marine sediment | 4 | 0.00 |
| CAM_SMPL_000832 | 97560 | Broadphages-iMicrobes | Virome Methanogenic sediments virome | marine sediment | 0 | 0.00 |
| CAM_SMPL_000833 | 223500 | Broadphages-iMicrobes | Virome Suboxic marine basin virome | seawater | 7 | 0.00 |
| CAM_SMPL_000835 | 147478 | Broadphages-iMicrobes | VASVAL242/1 | marine sediment | 2 | 0.00 |
| CAM_SMPL_000836 | 229553 | Broadphages-iMicrobes | Virome Great Boiling Spring virome | hot springs | 3 | 0.00 |
| CAM_SMPL_000837 | 257247 | Broadphages-iMicrobes | Virome_HOT219S2C13_F3 | seawater | 117 | 0.05 |
| CAM_SMPL_000838 | 208244 | Broadphages-iMicrobes | Virome induced lysogens 030409-2i | seawater | 0 | 0.00 |
| CAM_SMPL_000839 | 228819 | Broadphages-iMicrobes | Virome 13C-enriched ANME virome | sediment | 0 | 0.00 |
| CAM_SMPL_000840 | 266554 | Broadphages-iMicrobes | Virome Archaeal dominated cold seeps Costa Rica dsDNA virome | seawater | 1 | 0.00 |
| CAM_SMPL_000841 | 228925 | Broadphages-iMicrobes | Virome Archaeal dominated cold seeps Costa Rica ssDNA virome | seawater | 0 | 0.00 |
| CAM_SMPL_000842 | 224193 | Broadphages-iMicrobes | VAGALB1/1 | marine sediment | 0 | 0.00 |
| CAM_SMPL_000844 | 194325 | Broadphages-iMicrobes | Virome induced lysogens 030409-2a | seawater | 0 | 0.00 |
| CAM_SMPL_000845 | 104669 | Broadphages-iMicrobes | Virome BADE1 | seawater | 14 | 0.01 |
| CAM_SMPL_000846 | 185154.5 | Broadphages-iMicrobes | 12C Fraction Aerobic Sediment Virome | sediment | 1 | 0.00 |
| CAM_SMPL_000847 | 121688 | Broadphages-iMicrobes | Virome ME-08-3 | seawater | 184 | 0.15 |
| CAM_SMPL_000954 | 98103 | Broadphages-iMicrobes | Virome Appledore Island sediment RNA virome | marine sediment | 0 | 0.00 |
| CAM_SMPL_000956 | 267169 | Broadphages-iMicrobes | Virome Subarctic Pacific-4 | seawater | 7 | 0.00 |
| CAM_SMPL_000957 | 265601 | Broadphages-iMicrobes | Virome Subarctic Pacific-5 | seawater | 57 | 0.02 |
| CAM_SMPL_000958 | 241571 | Broadphages-iMicrobes | Virome 072/01/2.3m | seawater | 6 | 0.00 |
| CAM_SMPL_000959 | 184906 | Broadphages-iMicrobes | Virome Minimetagenomics 3 | seawater | 11 | 0.01 |
| CAM_SMPL_000960 | 150042 | Broadphages-iMicrobes | Virome Gulf of Maine 2 | seawater | 186 | 0.12 |
| CAM_SMPL_000961 | 272681 | Broadphages-iMicrobes | Uncultured virus Virus 2. San Pedro Ocean Time Series Microbial Observatory | seawater | 8 | 0.00 |
| CAM_SMPL_000964 | 262809 | Broadphages-iMicrobes | Virome Saanich Suttle Oxic-3 | seawater | 2 | 0.00 |
| CAM_SMPL_000966 | 245248 | Broadphages-iMicrobes | Viral metagenome ANOVIR Chesapeake Bay 4 | seawater | 19 | 0.01 |
| CAM_SMPL_000967 | 206564 | Broadphages-iMicrobes | multiple hosts NL18_2009-10 DNA | hot springs | 1 | 0.00 |
| CAM_SMPL_000969 | 224509 | Broadphages-iMicrobes | Uncultured virus NL17_2010-06-02 RNA viral metagenome | hot springs | 0 | 0.00 |
| CAM_SMPL_000970 | 276096 | Broadphages-iMicrobes | Virome Subarctic Pacific-9 | seawater | 88 | 0.03 |
| CAM_SMPL_000971 | 268557 | Broadphages-iMicrobes | Virome Saanich Suttle Anoxic-1 | seawater | 1 | 0.00 |
| CAM_SMPL_000973 | 213191 | Broadphages-iMicrobes | multiple hosts NL10_2010-02 RNA | hot springs | 4 | 0.00 |
| CAM_SMPL_000974 | 208207 | Broadphages-iMicrobes | Virome Gulf of Maine 1 | seawater | 11 | 0.01 |
| CAM_SMPL_000976 | 156657 | Broadphages-iMicrobes | multiple hosts NL17_2009-10 DNA | hot springs | 1 | 0.00 |
| CAM_SMPL_000977 | 298442 | Broadphages-iMicrobes | multiple hosts WTA NL 16 | hot springs | 4 | 0.00 |
| CAM_SMPL_000978 | 115015 | Broadphages-iMicrobes | multiple hosts WTA NL 18 | hot springs | 1 | 0.00 |
| CAM_SMPL_000979 | 296016 | Broadphages-iMicrobes | Virome Subarctic Pacific-10 | seawater | 3 | 0.00 |
| CAM_SMPL_000980 | 278821 | Broadphages-iMicrobes | Virome Minimetagenomics 4 | seawater | 0 | 0.00 |
| CAM_SMPL_000981 | 87274 | Broadphages-iMicrobes | Uncultured virus GOMss Achan-JL2 | seawater | 1 | 0.00 |
| CAM_SMPL_000982 | 252792 | Broadphages-iMicrobes | Virome Saanich Suttle Oxic-2 | seawater | 1 | 0.00 |
| CAM_SMPL_000983 | 154473 | Broadphages-iMicrobes | multiple hosts NL10_2010-02 DNA | hot springs | 0 | 0.00 |
| CAM_SMPL_000984 | 194474 | Broadphages-iMicrobes | Uncultured virus CHAS_2010-06-02 DNA viral metagenome | hot springs | 3 | 0.00 |
| CAM_SMPL_000985 | 344039 | Broadphages-iMicrobes | Virome Subarctic Pacific-2 | seawater | 9 | 0.00 |
| CAM_SMPL_000986 | 304409 | Broadphages-iMicrobes | Virome Subarctic Pacific-7 | seawater | 63 | 0.02 |
| CAM_SMPL_000987 | 300136 | Broadphages-iMicrobes | Virome 142/01/15.5m | seawater | 84 | 0.03 |
| CAM_SMPL_000988 | 202525 | Broadphages-iMicrobes | Virome Minimetagenomics 1 | seawater | 17 | 0.01 |
| CAM_SMPL_000989 | 221802 | Broadphages-iMicrobes | Virome Saanich Suttle Anoxic-2 | seawater | 2 | 0.00 |
| CAM_SMPL_000990 | 247306 | Broadphages-iMicrobes | Uncultured virus Virus 1. San Pedro Ocean Time Series Microbial Observatory | seawater | 144 | 0.06 |
| CAM_SMPL_000992 | 242045 | Broadphages-iMicrobes | Virome Suttle GOB5 | seawater | 0 | 0.00 |
| CAM_SMPL_000993 | 312563 | Broadphages-iMicrobes | Virome Saanich Suttle Oxic-1 | seawater | 1 | 0.00 |
| CAM_SMPL_000994 | 254852 | Broadphages-iMicrobes | Viral metagenome ANOVIR Chesapeake Bay 2 | seawater | 11 | 0.00 |
| CAM_SMPL_000996 | 48554 | Broadphages-iMicrobes | Virome Appledore Island water column RNA virome | seawater | 0 | 0.00 |
| CAM_SMPL_000997 | 185107 | Broadphages-iMicrobes | Uncultured virus NL18_2010-06-02 RNA viral metagenome | hot springs | 0 | 0.00 |
| CAM_SMPL_000998 | 332263 | Broadphages-iMicrobes | Virome Subarctic Pacific-3 | seawater | 0 | 0.00 |
| CAM_SMPL_000999 | 153852 | Broadphages-iMicrobes | Virome 020/01/20.3m | seawater | 55 | 0.04 |
| CAM_SMPL_001000 | 97781 | Broadphages-iMicrobes | Viral metagenome ANOVIR Chesapeake Bay 1 | seawater | 8 | 0.01 |
| CAM_SMPL_001003 | 331803 | Broadphages-iMicrobes | Virome Subarctic Pacific-6 | seawater | 20 | 0.01 |
| CAM_SMPL_001004 | 96950 | Broadphages-iMicrobes | Uncultured virus Saanichss Achan-JL3 | seawater | 1 | 0.00 |
| CAM_SMPL_001005 | 43366 | Broadphages-iMicrobes | Uncultured virus Virus 5. | seawater | 0 | 0.00 |
| CAM_SMPL_001006 | 205982 | Broadphages-iMicrobes | Virome Suttle GOB1 | seawater | 1 | 0.00 |
| CAM_SMPL_001008 | 123820 | Broadphages-iMicrobes | multiple hosts WGA NL 16 | hot springs | 0 | 0.00 |
| CAM_SMPL_001009 | 238668 | Broadphages-iMicrobes | multiple hosts WTA NL 17 | hot springs | 1 | 0.00 |
| CAM_SMPL_001010 | 240901 | Broadphages-iMicrobes | Uncultured virus CHAS_2010-06-02 RNA viral metagenome | hot springs | 0 | 0.00 |
| CAM_SMPL_001011 | 325857 | Broadphages-iMicrobes | Virome Subarctic Pacific-1 | seawater | 142 | 0.04 |
| CAM_SMPL_001012 | 337451 | Broadphages-iMicrobes | Virome Subarctic Pacific-8 | seawater | 34 | 0.01 |
| CAM_SMPL_001013 | 212996 | Broadphages-iMicrobes | Virome Minimetagenomics 2 | seawater | 107 | 0.05 |
| CAM_SMPL_001015 | 355400 | Broadphages-iMicrobes | Virome Helgoland after bloom virome | seawater | 81 | 0.02 |
| CAM_SMPL_001016 | 181000 | Broadphages-iMicrobes | Virome Suttle GOB Saanich Oxic | seawater | 0 | 0.00 |
| CAM_SMPL_001017 | 236433 | Broadphages-iMicrobes | Virome Suttle GOB2 | seawater | 0 | 0.00 |
| CAM_SMPL_001171 | 238096 | Broadphages-iMicrobes | Uncultured virome Virome 2 | seawater | 7 | 0.00 |
| CAM_SMPL_001173 | 12368 | Broadphages-iMicrobes | Uncultured virome Virome 3 | seawater | 0 | 0.00 |
| CAM_SMPL_001174 | 214038 | Broadphages-iMicrobes | Uncultured virome Virome 1 | seawater | 0 | 0.00 |
| CAM_SMPL_A0003 | 231246 | Broadphages-iMicrobes | G2810 | seawater | 48 | 0.02 |
| 41 | 93744 | Metavir | Kingman | seawater | 10 | 0.01 |
| 44 | 279882 | Metavir | Kiribati | seawater | 11 | 0.00 |
| 42 | 318178 | Metavir | Palmyra | seawater | 24 | 0.01 |
| 43 | 378475 | Metavir | Tabueran | seawater | 17 | 0.00 |
| 4808 | 2051566 | Metavir | Coral larvae exposure (GWR) | Coral | 0 | 0.00 |
| 4682 | 163070 | Metavir | PoC3TA | Coral | 16 | 0.01 |
| 2943 | 1706540 | Metavir | A. tenuis DNA | Coral | 130 | 0.01 |
| 2944 | 734031 | Metavir | A. tenuis RNA SISPA | Coral | 9 | 0.00 |
| 2698 | 370135 | Metavir | P. damicornis CFM repliG | Coral | 1 | 0.00 |
| 2696 | 292476 | Metavir | P. damicornis LN2 repliG | Coral | 3 | 0.00 |
| 2711 | 2000000 | Metavir | P. damicornis LN2 SISPA | Coral | 121 | 0.01 |
| 2694 | 435494 | Metavir | P. damicornis NLN repliG | Coral | 12 | 0.00 |
| 2712 | 2000000 | Metavir | P. damicornis NLN SISPA | Coral | 433 | 0.02 |
| 815 | 140645 | Metavir | A. hycinthus 2010 | Coral | 0 | 0.00 |
| 814 | 67845 | Metavir | A. hycinthus 2012 | Coral | 0 | 0.00 |
| 816 | 181031 | Metavir | A. millepora 2010 | Coral | 1 | 0.00 |
| 817 | 95400 | Metavir | A.millepora 2012 | Coral | 1 | 0.00 |
| 965 | 403686 | Metavir | A. millepora post-bleach | Coral | 2 | 0.00 |
| 964 | 401070 | Metavir | A. millepora pre-bleach | Coral | 1 | 0.00 |
| 818 | 88020 | Metavir | A. palmata 2011 | Coral | 0 | 0.00 |
| 819 | 89190 | Metavir | A. tenuis 2012 | Coral | 2 | 0.00 |
| 702 | 930 | Metavir | D. strigosa bleach | Coral | 0 | 0.00 |
| 699 | 1580 | Metavir | D.strigosa healthy | Coral | 0 | 0.00 |
| 820 | 2173 | Metavir | M. annularis | Coral | 0 | 0.00 |
| 1139 | 104166 | Metavir | M. cavernosa stressed | Coral | 2 | 0.00 |
| 1138 | 56218 | Metavir | M. cavernosa control | Coral | 0 | 0.00 |
| 821 | 21096 | Metavir | M. faveolata | Coral | 0 | 0.00 |
| 2315 | 71335 | Metavir | P. acuta 2012 | Coral | 0 | 0.00 |
| 822 | 92142 | Metavir | P. astreoides 2010 | Coral | 1 | 0.00 |
| 813 | 50205 | Metavir | P. astreoides 2012 | Coral | 0 | 0.00 |
| 835 | 295437 | Metavir | P. astreoides prokaryotes | Coral | 3 | 0.00 |
| 2341 | 905686 | Metavir | P. damicornis base | Coral | 12 | 0.00 |
| 3360 | 3135 | Metavir | Mad1 | Coral | 0 | 0.00 |
| 3359 | 4382 | Metavir | Mus1 | Coral | 2 | 0.05 |
| 1478 | 320104 | Metavir | GS108 | seawater | 85 | 0.03 |
| 1477 | 494832 | Metavir | GS112 | seawater | 130 | 0.03 |
| 1479 | 480375 | Metavir | GS117 | seawater | 279 | 0.06 |
| 1480 | 341386 | Metavir | GS122 | seawater | 45 | 0.01 |
| 3665 | 3344 | Metavir | GS122_assembly | seawater | 0 | 0.00 |
| 8071 | 127855 | Metavir | jeju12clc | seawater | 24 | 0.02 |
| 8050 | 233762 | Metavir | jeju3clc | seawater | 42 | 0.02 |
| 8051 | 167416 | Metavir | jeju6clc | seawater | 7 | 0.00 |
| 8070 | 190864 | Metavir | jeju9clc | seawater | 61 | 0.03 |
| 8084 | 159503 | Metavir | south Pacific 12clc | seawater | 37 | 0.02 |
| 8076 | 83812 | Metavir | south Pacific 3clc | seawater | 29 | 0.03 |
| 8077 | 84784 | Metavir | south Pacific 6clc | seawater | 17 | 0.02 |
| 8083 | 143798 | Metavir | south Pacific 9clc | seawater | 27 | 0.02 |
| 2895 | 3210 | Metavir | Dunk Island contigs | seawater | 1 | 0.03 |
| 1357 | 116855 | Metavir | Dunk Island_F1 | seawater | 64 | 0.05 |
| 2896 | 2382 | Metavir | Fitzroy Island contigs | seawater | 4 | 0.17 |
| 1358 | 82739 | Metavir | Fitzroy Island_F1 | seawater | 41 | 0.05 |
| 3816 | 11189 | Metavir | ALOHA station deep | seawater | 0 | 0.00 |
| 3815 | 7838 | Metavir | ALOHA station upper euphotic | seawater | 0 | 0.00 |
| 13 | 262501 | Metavir | Gulf of Mexico | seawater | 1 | 0.00 |
